# ‘Perceptions of Performance Appraisal Quality’ and Employee Innovative Behavior: Do Psychological Empowerment and ‘Perceptions of HRM System Strength’ Matter?

**DOI:** 10.3390/bs8120114

**Published:** 2018-12-15

**Authors:** Aamer Waheed, Qaisar Abbas, Omer Farooq Malik

**Affiliations:** 1 Department of Management Sciences, COMSATS University Islamabad, Park Road, Tarlai Kalan, Islamabad 45550, Pakistan; omer_farooq@comsats.edu.pk; 2 Director, COMSATS University Islamabad, Lahore Campus, Lahore 54000, Pakistan; qaisar@comsats.edu.pk

**Keywords:** innovative behavior, psychological empowerment, perceptions of performance appraisal quality, perceptions of HRM system strength, Pakistan

## Abstract

Organizations need to be innovative for their long-term survival and this can be achieved when their employees demonstrate innovative behaviors at the workplace. Innovative behavior has thus received considerable attention from researchers, particularly on exploring the factors which foster employee innovative behaviors. Based on human resource system strength theory, the objective of this study is twofold. First, it examines the direct and indirect relationship between perceptions of performance appraisal quality (PPAQ) and innovative behavior mediated through psychological empowerment. Second, it examines the moderating effect of perceptions of HRM system strength in the hypothesized links. A total of 360 faculty members participated in the study from twelve public sector higher education institutes in Islamabad, Pakistan. Partial least squares structural equation modeling (PLS-SEM) is used for statistical analysis of the quantitative data collected through self-administered questionnaire. Results demonstrated that PPAQ is positively related with innovative behavior. The findings also support the mediating role of psychological empowerment and the moderating role of perceptions of human resource management (HRM) system strength. We contribute to the literature by demonstrating that HRM content and process are two complementary facets of an HRM system in bringing out positive work behaviors. A number of practical implications and directions for future research are outlined.

## 1. Introduction

In a knowledge-based economy, innovation is vital to organizational success by developing, implementing, and promoting new knowledge, ideas, technology, and business models [1,2,3]. The inspiration of an individual plays a pivotal role in the development of new products and/or services [4]. Innovative employees keep on searching for new opportunities to satisfy their creative appetites [5]. The innovative behavior of employees is recognized as the foundation for organizational innovation [6] and competitive advantage [7,8] where the employees’ capability of being innovative in their methods, goods, and services sets the basis for development [9]. Therefore, innovative behavior has become imperative for performance improvement, organizational effectiveness, and success through innovative output [5,10,11]. Innovative behavior was initially recognized as an extra-role behavior [12,13] but now it is recognized as an in-role behavior because it has become a part of routine work [14,15]. The seminal work conducted by Katz [16,17] leads us in developing an understanding of innovative work behavior. According to Katz [16], innovative behavior is not a formal action of individuals that is used to deal with uncertainties and contingencies. The organization depends on the blueprints of their employees’ innovative behavior in a delicate social system. This description of organizational environment provided by Katz [16] does not fully address the rapid change and development in the business environment. The latest studies have succeeded in collecting pieces of evidence and acknowledged the importance of innovative behavior for organizational performance [17]. 

Innovative behavior is an intentional behavior of an employee that can be initiated within the individual’s work role, group, or organization to create and implement innovative ideas [18,19]. After two decades of Katz’s [16,17] propositions, Kanter [20] presented a model for innovative behavior that comprises of innovative activities or behaviors at the micro-level. The author suggested that idea generation, coalition building, idea realization and diffusion are the main components of innovative work behavior. Innovative behavior is the result of collaborative activities in which individuals are involved in creating, presenting, testing, and implementing the new ideas [20]. Innovative behavior is the outcome of the individual’s interaction with the present situation and providing a solution based on previous knowledge or experience [15,21]. Dorenbosch et al. [14] argue that innovative behavior is the invention and implementation of a new idea. Janssen [15] define innovative behavior as “the extent to which employees behave to create, promote, and implement new ideas in a group or organization.” Innovative behavior is the individual’s ability to reach an idea or a solution for a complex situation or problem faced by the organization [15]. In the present study, innovative behavior is defined as the process of generating novel solutions to problems; promoting the ideas by convincing colleagues; and implementing them within a group, unit or organization [3,21]. 

Capitalizing on innovative potential brings development and newness in products, services, and processes [22]. Organizations should create an atmosphere to promote innovation and encourage employees to be creative and innovative in their work [23]. Organizations should develop cultures where innovative behavior is practiced and knowledge is used in an imaginative way. Moreover, employees should be willing to participate in innovative processes on a continuous basis [22]. Consequently, it becomes important for organizations to understand the factors that help in generating and promoting innovative behavior [10]. Underscoring the importance of employee innovative behavior, several studies have examined its antecedents such as job characteristics [24], leadership [25,26] organizational structures [27], job satisfaction [28], organizational commitment [29], procedural justice [30], and supervisory support [31]. 

A large body of knowledge has contributed to understanding how various human resource management (HRM) practices may result in positive organizational outcomes, including innovation [32,33]. A number of studies have linked various HRM practices with innovative behavior such as training and development [34,35], rewards [5], job security [36], autonomy [37], task composition [14], job demands [38], and feedback [39]. Although it has been concluded that HRM practices enhance innovative behavior among employees, few studies have focused on the joint effect of HRM content, i.e. perceptions of performance appraisal quality (PPAQ) and HRM process i.e. perceptions of HRM system strength. Performance appraisal is an important HR practice and is recognized as a motivating factor that provides quality feedback and encourages employees to take initiative in their work [40]. Bednall et al. [41] suggested that PPAQ (as a content of HRM) is positively linked with employee innovative behavior. However, they did not investigate the psychological mechanism that may be responsible for transmitting the effect of PPAQ on innovative behavior. 

HR system strength (HRSS) theory [42] proposes that HRM in terms of content (i.e. specific HRM practice) and process (i.e. perceptions of HRM system strength) create a psychological climate which in turn influences employees’ attitudes and behaviors. The theory also posits that psychological empowerment as a positive job attitude plays a vital role in shaping the behaviors of individuals in the workplace [42,43]. Thus, it becomes imperative to examine the mediating role of psychological empowerment in the relationship between PPAQ and employee innovative behavior. The performance appraisal process provides guidance functions to employees because when they perceive quality in performance appraisals, they may feel psychologically empowered to take initiatives and illustrate innovative behaviors. Based on HRSS theory [42], in particular, this study 1) examines the relationship between PPAQ and innovative behavior, 2) investigates the mediating role of psychological empowerment in the relationship between PPAQ and innovative behavior, and 3) determines whether perceptions of HRM system strength moderate the effects of PPAQ on psychological empowerment and innovative behavior.

## 2. Literature Review and Hypotheses Development

### 2.1. Perceptions of Performance Appraisal Quality and Innovative Behavior

Performance appraisal is an important HRM practice [13] because it motivates employees to display attitudes and behaviors that are consistent with the organizational strategy [40]. Employees’ reactions to performance appraisals are critical because the fairness and quality of the appraisal lead to desired attitudes and behaviors [44,45]. Employees are sensitive to the quality of performance appraisals because an ineffective or low-quality performance appraisal leads to negative outcomes [46,47]. The quality of the performance appraisal depends on the feedback from the supervisor and the delivery mechanism [47,48]. 

Bednall et al. [41] defined PPAQ in terms of its clarity, regularity, and openness. Employees perceive the quality in the feedback when the supervisor regularly holds appraisal meetings, clearly communicates the feedback, and openly discusses the problems [41]. Clarity refers to the employees’ familiarity with the performance appraisal process in terms of its purpose and their role. It comprises of performance expectation and provides the correct information regarding the current and desired performance during the feedback [47]. Regularity indicates the pattern of the ongoing feedback. The employee should engage with performance feedback at regular intervals to judge their performance and make improvements. Finally, openness refers to the level of communication in which the employees and the supervisor share their views and feedback on performance appraisal [47]. Literature suggests that the organization should encourage open dialogue in the feedback process [49].

Research has revealed that those employees who perceive quality in performance appraisal demonstrate positive attitudes and behaviors such as increased job satisfaction, affective commitment, and work performance [47,50,51,52]. Moreover, they are less likely to quit their job [47,53]. The link between PPAQ and its outcomes can be explained by utilizing HRSS theory [42]. HRSS theory posits that the employees’ perceptions of HRM practices influence their attitudes and behaviors. It communicates to employees about the expected behaviors that are rewarded [42]. It also helps organizations to maximize the performance of employees by facilitating positive reactions at work [54]. It is argued that performance appraisal quality improves employees’ feelings of achievement and helps them to understand their role in the organization [55]. In support of these arguments, Bednall et al. [41] found that PPAQ is positively linked with innovative behavior. It can thus be argued that employees who perceive quality in performance appraisal are more innovative in their work. Therefore, we hypothesize the following:

**Hypothesis** **1.** *Perceptions of performance appraisal quality have a positive effect on the innovative behavior of employees.*

### 2.2. Mediating Role of Psychological Empowerment

Two theoretical views on empowerment are found in the literature, i.e. social-structural empowerment and psychological empowerment [56,57,58]. Social-structural empowerment is based on contextual factors and social structures in the workplace at the macro level. On the other hand, psychological empowerment focuses on cognition or psychological perspective on the micro level [58]. Thus, structural empowerment focuses on how work has to be done while psychological empowerment emphasizes how employees experience their work. Psychological empowerment has evolved over time, leading to various schools of thought such as Conger and Kanungo [59], Thomas and Velthouse [60] and Spreitzer [57]. In their views, psychological empowerment is the psychological responses or cognitive inspirations of individuals’ in response to organizational approaches and practices.

Conger and Kanungo [59] were the first to propose the psychological perspective of empowerment based on cognitions and emotions of individuals or self-efficacy beliefs. They defined psychological empowerment as a process which helps in enhancing feelings of self-efficacy among employees. In their view, empowerment is not only the delegation of authority and power to the subordinate, but a personal belief of an employee about his/her role and relationship with the organization [58]. According to Conger and Kanungo [59], the employee self-efficacy can be enhanced by delegating the authority and resource sharing. Psychological empowerment is understood as when the employees are given authority, autonomy, and freedom in carrying out their work. Thomas and Velthouse [60] presented a cognitive model of empowerment in which psychological empowerment is shaped by individual work contexts and personality traits. Empowerment is not a dispositional trait, but the manifestation of four cognitions of intrinsic task motivation. Thomas and Velthouse [60] define empowerment as “a set of cognitions or states influenced by the work environment that helps and create an active-orientation to one’s job” [58]. The core of Thomas and Velthouse [60] and Conger and Kanungo [59] are the intrinsic task motivation of employees known as self-efficacy beliefs consisting of four cognitions i.e. meaning, competence, choice, and impact. 

Spreitzer [57] defines psychological empowerment as the reflection of individual’s intrinsic task motivation or orientation of work role. It consists of the cognitions shaped by the environment rather than fixed attributes of personality. According to Spreitzer, psychological empowerment is the mindfulness and an obligation-based design which requires that an individual is actively orientated to work role. Furthermore, the work environment shapes the cognitions of the employee. This is contrary to the belief that a fixed personality attribute shapes the cognitive aspect of the employee. It is a psychological condition or feeling of competence, meaning, impact, and self-determination [57]. Competence is the employees’ feelings of their abilities to deal with problems and obstacles. Meaning refers to the importance given by an employee to a job task he/she is performing. Employees who give meaning to their work are more committed and put in more effort [61]. Impact refers to employees’ feeling of their influence in tasks and outcomes at work [57]. Finally, self-determination is employees’ feeling of autonomy or persistence and flexibility in adapting the changing situation. In the present study, we follow the conceptualization of psychological empowerment as provided by Spreitzer [57].

Based on HRSS theory [42], we argue that performance appraisal is an important HR practice and PPAQ is considered a crucial content of the HRM system which sends signals, communicates promises, and psychologically empowers employees to obtain the desired contributions [62]. Previous research has demonstrated that high performance managerial practices are positively related to psychological empowerment [63]. In addition, Aryee et al., [33] found a significant positive relationship between high performance work practices and psychological empowerment. Similarly, Messersmith et al., [64] also demonstrated the positive influence of high performance work system on psychological empowerment. Therefore, it can be argued that PPAQ leads employees to feel empowered.

Further, we theorize that psychological empowerment has a direct positive influence on employee innovative behavior. Psychological empowerment allows employees to realize their potential and thus be innovative in their work, groups or organization [65]. According to psychological empowerment theory [58], empowered employees take an active orientation towards their work which facilitates innovative behavior [66]. The psychologically empowered employees act independently from the other. They feel competent and influential to their work by showing initiative and proactive behaviors [8,56,57,60]. Previous research shows that the performance of empowered employees is superior to those who are less empowered [61]. It is also evident that psychological empowerment of employees plays a vital role in developing and promoting innovative behavior among employees [57,63]. For example, Knol & Linge, [39] found that innovative behavior is positively influenced by psychological empowerment.

The quality of performance appraisal enhances the perceived obligations on the part of employees towards their organization which results in positive attitudes and behaviors [47,52]. Spreitzer [57] argues that the work context promotes innovative behavior by psychologically empowering employees. In line with the HRSS theory [42], it can be argued that other than having a direct relationship, positive perceptions regarding an HRM practice (e.g. PPAQ) have an indirect relationship with innovative behavior through the mediation of psychological empowerment. Thus, we hypothesize the following:

**Hypothesis** **2.** *Psychological empowerment mediates the relationship between perceptions of performance appraisal quality and innovative behavior.*

### 2.3. Moderating role of Perceptions of HRM System Strength

Three distinct conceptualizations of perceptions of HRM system strength are found in literature. First, Bowen and Ostroff [42] explained the difference between the content and process of HRM. The content refers to the individual HR practice or set of practices intended to achieve specific objectives whereas, the process refers to the structural support and processes followed by the HR department. According to them the HRM-performance linkage can be better understood by keeping in mind the strength of the HRM system. The role of the HR department is critical in this regard as the development and implementation of HR policies is the responsibility of the HR department [67]. The HRM system is stronger when the messages are properly communicated and the policies are properly implemented by line managers in accordance with the prescribed plans [68,69]. The HRM system communicates the requirements to employees regarding their performance. It sends signals to employees about the expected behavior that is rewarded for the achievement of organizational goals [42,68,69]. In addition, the strong system is characterized by having high levels of distinctiveness, consistency, and consensus in the messages communicated by the HR department [42,70,71]. 

Second, HRM system strength is conceptualized as the perception of an employee regarding the bundle or set of practices implemented. A high number of practices implies more signals from the HR department to an employee about his/her performance [72]. However, this does not mean that the practices are distinctive and consistent. Moreover, the higher number of practices does not ensure censuses among the policy makers. Third, HRM system strength is conceptualized as the ratings on content of HRM i.e. specific HR practice. A higher rating on a particular practice indicates a strong system as the ratings justify the shared perceptions of a stronger system among employees [73,74]. These two definitions of HRM system lack focus on the three meta-features as proposed by Bowen and Ostroff [42], which are based on the co-variation principle of attribution theory [75]. Within HRSS theory, it is mandatory that the organization must provide distinct, consistent, and consensual HR messages at all levels. Clear, consistent, and unambiguous messages create perceptions of a strong HR system, which in turn lead to a strong organizational climate [42,76]. The strong climate generated through a strong system motivates employees to demonstrate positive attitudes and behaviors [42,68,69].

According to Bowen and Ostroff [42], distinctiveness refers to the delivery of visible, understandable, legitimate, and relevant messages to employees for their attention. Consistency refers to the articulation of HR practices in such a way that it appears reliable and coherent across different levels of the organizational hierarchy at a different time. Consistency is the establishment of a constant relationship with individuals and the environment over time [42]. It is the understanding of cause and effect over time and implies that when some event occurs, the cause of that event also exists [77]. The consistency in messages informs the employees about the specific behaviors that are expected and rewarded in the workplace [30]. Finally, consensus refers to the agreement or generalization of an incident among all individuals [42,71]. It is important that individuals perceive the same effect and fairness in their treatment [42].

Previous research has demonstrated that employees’ perceptions of the HRM system tend to have a positive impact on affective commitment [78,79], job satisfaction [71], motivation [80], and work performance [81,82]. It also enhances employees’ perceptions of organizational support towards their work and goal attainment [83]. Moreover, the perceptions of HRM system strength tend to reduce job strain [84], emotional exhaustion [85], and intention to quit [86]. The HRSS theory [42] has been applied in different areas and particularly in HR research [70], where the focus of the studies was on exploring the role of the HRM system in explaining employees’ responses to different HRM practices [87]. The question regarding how a particular HRM practice leads to desired organizational outcomes can better be understood through the combination of both the content and the process of HRM. The key is to investigate how the HRM system is perceived by the employee and stimulates desired attitudes and behaviors. A major premise of HRSS theory is that HR practices influence individual level attitudes and behaviors where perceptions of HRM system strength, moderate these linkages [42,62,87]. Based on these arguments, Bednall et al. [41] found that the positive relationship between PPAQ and innovative behavior was strengthened by positive perceptions of HRM system strength. Therefore, it is argued that employees who perceive strengths in the HRM system feel more psychologically empowered and demonstrate innovative behavior. This leads us to the following hypotheses:

**Hypothesis** **3a.** *Perceptions of HRM system strength moderate the positive relationship between perceptions of performance appraisal quality and psychological empowerment.*

**Hypothesis** **3b.** *Perceptions of HRM system strength moderate the positive relationship between perceptions of performance appraisal quality and innovative behavior.*

### 2.4. The Present Study

**Figure 1:**
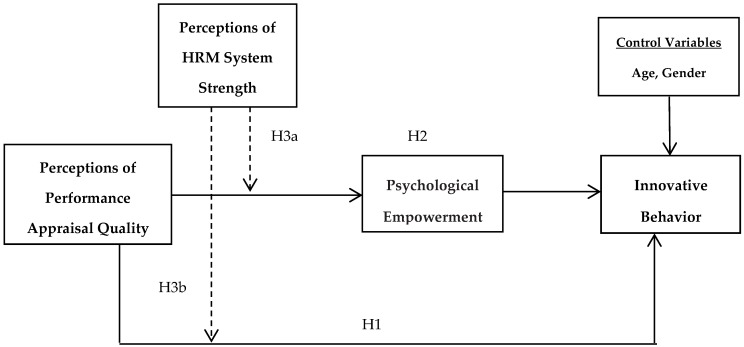
Research model.

The research model is presented in Figure 1. The research model posits that PPAQ positively influences psychological empowerment that, in turn, positively influences employee innovative behavior. Furthermore, the positive effects of PPAQ on psychological empowerment and innovative behavior are strengthened by positive perceptions of HRM system strength.

In the present study, we focused on faculty members working in public sector higher education institutes (HEIs) in Pakistan. The faculty of HEIs is acknowledged as being the facilitators of innovation in Pakistani society by demonstrating innovative behavior [10,22]. The HEIs are supposed to beat the competition, overcome barriers, and tackle resistance to change [88]. In other words, HEIs keep changing their structure, functions, and governance to meet the changing demands of their stakeholders and society [89]. 

In the last two decades, the educational institutions have been involved in embedding innovation in their academic system [90]. Following this trend, national level reforms and huge investments have been made for innovation and human development of HEIs in Pakistan [91]. The Higher Education Commission, Pakistan (HEC) aims to continue the trend through faculty development, quality improvement, promoting innovation, and integrating with the society. The establishment of the Offices of Research, Innovation and Commercialization (ORIC), Small Business Innovation Research (SBIR), and Business Incubation Centre (BIC) are a few initiatives to promote research and innovation in Pakistani HEIs [92]. The understanding of innovation by the faculty members is crucial in developing sustainability competencies in HEIs [88]. Thus, the HEIs in Pakistan are considered to be a suitable context in which the relationship can be studied among with the variables. 

## 3. Methodology

### 3.1. Participants

Data were collected from full-time faculty members who were involved in teaching and research in twelve public sector HEIs located in the federal capital of Islamabad, Pakistan. The sample held the positions of research associates, lecturers, assistant professors, associate professors and professors from a variety of academic departments. The demographic information was collected on the respondent’s age, gender, current job position, education level, and tenure. In a sample 59.3% of the participants were male and 40.7% were female. In terms of job position the sample of faculty members was established from 25 research associates (7%), 183 lecturers (51%), 132 assistant professors (37%), 16 associate professors (4%) and 4 professors (1%). From the respondent’s qualification, 211 held a master’s degree (58.6%), 102 were held a doctoral degree (28.3%) and 47 possessed a post-doctoral qualification (13.1%). In terms of age, 53.6% were 30–40 years of age. The tenure shows that 55% had between 1–5 years of service in their present organization and 33% had in 6–10 years of service.

### 3.2. Procedure

Self-administered questionnaire was used to collect the data from faculty members of HEIs by using convenience sampling. In total, 600 questionnaires were distributed among faculty members and 377 questionnaires were filled and returned, with a response rate of 63%. Of these, 360 responses were deemed appropriate for further data analysis. The data collection method followed a strict adherence to ethical guidelines. The administrations of sampled universities were informed about the objectives of the study and a copy of questionnaire was also provided to them. The respondents were also assured of confidentiality and anonymity. 

### 3.3. Measures

A 5-point Likert scale was used to measure all the variables. The anchors ranged from 1 (strongly disagree) to 5 (strongly agree). 

The 5-item scale of Bednall, Sanders and Runhaar [41] was used to measure innovative behavior of employees. A sample item is “I promote and defend my innovative ideas to others.” Composite reliability (CR) was 0.924.

The PPAQ was measured using the scale developed by Bednall et al. [41]. The 3-item scale was intended to assess the clarity, regularity and openness. A sample item is “In performance appraisals I get clear feedback on my performance.” CR was 0.912.

A 16-item scale developed by Delmotte, Winne, and Sels [71] was used to measure the perceptions of HRM system strength in regards to composite distinctiveness, consistency and consensus. A sample item for distinctiveness is “The procedures and practices developed by HR are easy to understand.” CR was 0.938.

A 12-item scale developed by Spretizer [57] was used to measure psychological empowerment as a composite of meaning, competence, self-determination and impact. A sample item is “My job activities are personally meaningful to me.” CR for the overall scale was 0.937.

The present study also included the demographics (age and gender) as covariates in the analysis to eliminate the alternative effect. The literature [15,31,41] suggest to control for these variables in relationships with innovative behavior. Age was measured on a 6-point ordinal scale, ranging from 1 (20–24 years) to 6 (45 years and above) and gender was measured as (0 = male, 1 = female).

## 4. Data Analysis

Two steps approach is followed in current study for the data analysis and estimation by using SmartPLS v.3.2.7 [93]. In the first step, the measurement model or outer model with reflective measures is assessed for indicator’s reliability and validity. The adequate support of the measurement model allows the assessment of the structural model or inner model in the second step. PLS has various strengths that made it suitable for data analysis, including its soft distributional assumptions, its flexibility in modeling higher-order constructs, and its ability to handle complex research models such as the combination of mediating and moderating effects [94].

### 4.1. Results

#### 4.1.1. Common Method Variance 

Common method variance can be a problem when single-source data is used to measure latent variables. To address this problem, we performed Harman single-factor test [95] on nine first-order latent variables in our research model. The result shows the emergence of more than one factor. The common method factor accounted for well below the 50% threshold variance. This indicated that the common method bias does not pose a significant problem with respect to collected data.

#### 4.1.2. Measurement Model

In a reflective measurement model of present study there were nine latent variables (perceptions of performance appraisal quality, meaning, competence, self-determination, impact, distinctiveness, consistency, consensus, and innovative behavior). The psychological empowerment and HRM system strength were measured, as composite variable and duplicate items were used for measurement purposes. Confirmatory factor analysis (CFA) was performed to evaluate the measurement model before the hypothesis testing. The composite reliability (CR) estimates are used to examine the internal consistency reliability. The CR value is well above the threshold level of 0.70 [96] which demonstrated a high level of internal consistency and reliability. The CR value is presented in Table 1. 

Convergent validity was assessed through the value of the average variance extracted (AVE) and factor loadings. The AVE value was well above the threshold level of 0.50 [96] and the items factor loading on their respective constructs were also above the threshold value of 0.70 [97]. The results provided support for convergent validity and are presented in Table 1. Discriminant validity shows that the constructs are different from each other. We followed the Fornell-Larcker [98] guidelines to test the discriminant validity. According to the Fornell-Larcker [98] criterion, the square root of the average variance extracted (AVE) for a construct should be greater than the correlation with other constructs in the model. As presented in Table 2, the square roots of the AVEs for all constructs are higher than the correlation of these constructs with other variables in the path model. These results implied support for discriminant validity.

#### 4.1.3. Structural Model

**Figure 2:**
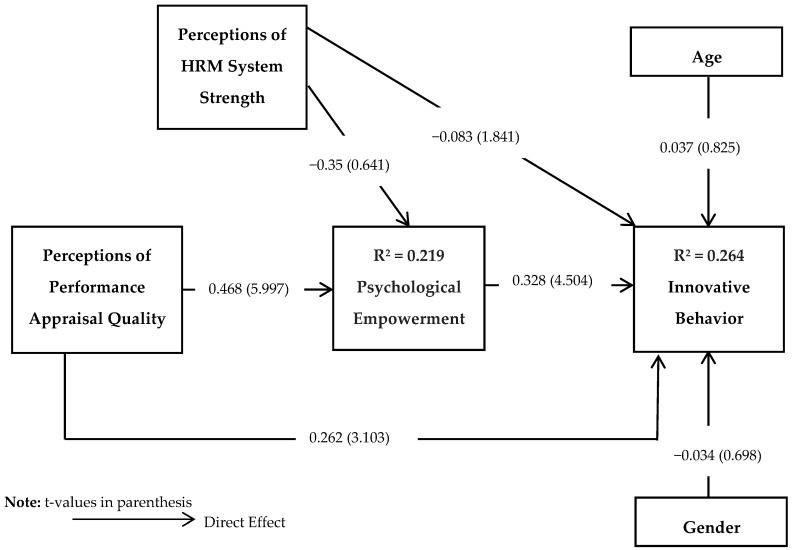
Main Effects Model.

The R^2^ value shows the predictive power or variance in endogenous variable explained by exogenous variables. The R^2^ value of 0.19, 0.33 and 0.67 in PLS models are assumed as weak, moderate and substantial. As shown in Figure 2, the R^2^ value estimated for psychological empowerment (mediating variable) and innovative behavior were 0.219 and 0.264 respectively, suggesting 21.9% and 26.4% of the variance explained by exogenous variables. Together, the results implied a satisfactory and substantial model. 

To test the proposed hypotheses the t-value is calculated with the process of bootstrapping. A 1000 bootstrap re-samples were used in estimation using *t-tests*. Bootstrapping is recognized as the latest techniques to determine the significance of path coefficient with estimates of t-value. Figure 2 shows the path coefficient and t-values. The examination of path coefficient’s results shows that PPAQ has a direct and positive impact on innovative behavior (*β* = 0.262; *t* = 3.103; *p* < 0.01). The results provided support for the acceptance of Hypothesis 1. The results for control variables (age, gender) demonstrated the non-significant effects on innovative behavior (see Figure 2). 

Indirect effect was examined using bootstrapping and 1000 bootstrap re-sampling was used to calculate the significance of the indirect effect [99,100]. The point estimate of 0.153 for the indirect effect at 95% bias-corrected confidence interval (0.078; 0.265) indicated that psychological empowerment mediates the relationship between PPAQ and innovative behavior. Because zero was not included in the confidence interval, thus it can be said that the indirect effect is significantly different from zero at *p* < 0.05, supporting Hypothesis 2. 

**Figure 3:**
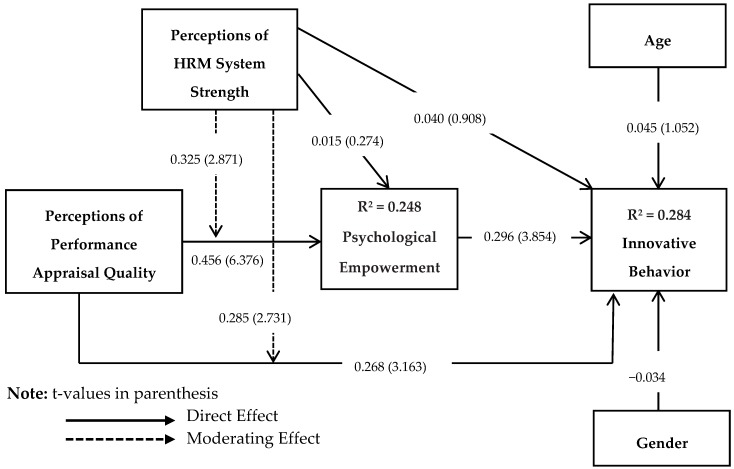
Interaction Effect Model.

**Figure 4:**
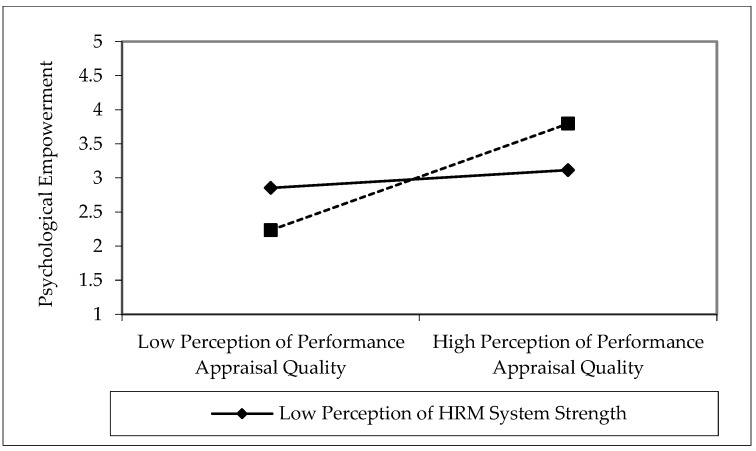
Interaction effect of perceptions of HRM system strength on perceptions of performance appraisal quality and psychological empowerment.

**Figure 5:**
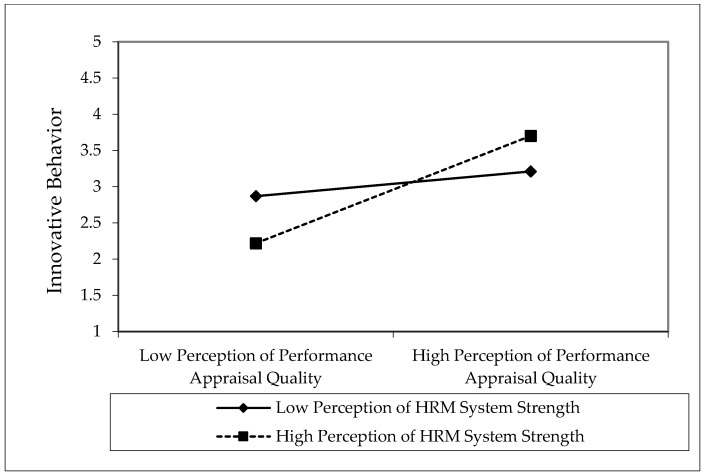
Interaction effect of perceptions of HRM system strength on perceptions of performance appraisal quality and innovative behavior.

Finally, we applied PLS product indicator approach [101] to test Hypotheses 3a and 3b. As shown in Figure 3, the interaction between PPAQ and perceptions of HRM system strength was positively related to psychological empowerment (*β* = 0.325; *t* = 2.871; *p* < 0.05). Similarly, the interaction between PPAQ and perceptions of HRM system strength was positively related to innovative behavior (*β* = 0.285; *t* = 2.731; *p* < 0.05). The interaction terms achieved the effect size (*f^2^*) values of 0.039 (psychological empowerment) and 0.021 (innovative behavior). The effect size (*f^2^*) of 0.02, 0.15, and 0.35 represents small, medium and large effect an exogenous variable has on endogenous variable [102]. The nature of interaction effects has been depicted by determining the 1SD slopes. The interaction pattern above and below the mean are shown in Figure 4 and Figure 5. The interactions show that PPAQ had a stronger (weaker) positive relationship with psychological empowerment (H3a) and innovative behavior (H3b) when the perceptions of HRM system strength were high (low). Thus, Hypothesis 3a and 3b were supported for moderating role of perceptions of HRM system strength. 

## 5. Discussion

The purpose of this study was to examine the direct and indirect effects of PPAQ on employee innovative behavior mediated through psychological empowerment. The study also examined the moderating role of perceptions of HRM system strength on the effects of PPAQ on psychological empowerment and innovative behavior. We demonstrated that PPAQ has a direct positive impact on innovative behavior. This finding supports the claim that HRM practices play an active role in shaping the behavior of employees in the workplace [41,47,65]. Further, we demonstrated that the relationship between PPAQ and innovative behavior was mediated by psychological empowerment. This finding is consistent with previous research e.g. references [33,63,64] which examined the mediating effect of psychological empowerment in the relationship between various HR practices and employee behaviors. The study also demonstrated that perceptions of HRM system strength moderate the positive effects of PPAQ on both psychological empowerment (mediator) and innovative behavior (dependent). These results are consistent with previous studies e.g. reference [41], which have found support for the moderating role of perceptions of HRM system strength on the relationship between PPAQ and innovative behavior. 

### 5.1. Theoretical Contributions

The current study makes several theoretical contributions. First, the study established an empirical link between PPAQ (HRM content) and employee innovative behavior. Thus, this study confirms the findings of Bednaal et al. [41] who also demonstrated the link between PPAQ and innovative behavior and suggested for future researchers to replicate the results of their study in other contexts and samples. Second, HRSS theory [42] posits that the effects of HRM practices on employee behaviors are direct as well as indirect mediated through positive job attitudes. The current study while lending support to this particular notion of HRSS theory demonstrated that psychological empowerment is an important underlying mechanism that is responsible for linking PPAQ and employee innovative behavior. As per our knowledge this is first of its kind to investigate the mediating role of psychological empowerment in underlying relationship. Thus, PPAQ makes employees feel empowered and as a result they become innovative in the workplace. Third, this study contributed to the literature by establishing the moderating role of perceptions of HRM system strength–a HRM process-in the relationship between PPAQ and psychological empowerment. We also found support for the moderating role of perceptions of HRM system strength in the relationship between PPAQ and innovative behavior. In particular, no study to the best of our knowledge has investigated the moderating role of perceptions of HRM system strength in relationship between PPAQ and psychological empowerment. By doing so, we succeeded in revealing that perceptions of HRM system strength play an important role in strengthening the positive relationship between PPAQ and psychological empowerment. Lastly, previous studies e.g. references [33,64,66] have established the relationship between HRM practices and several job attitudes and behaviors. However, in the majority of these studies the HRM practices were taken as a bundle or a set of practices, which made it impossible to determine the impact of a particular HRM practice (e.g. performance appraisal) on innovative behavior. This important gap in the literature was filled by the current study by examining and partialling out the impact of PPAQ on innovative behavior mediated through psychological empowerment. 

### 5.2. Practical Implications

The results of the study have some practical implications for multiple stakeholders, including employees, managers, and policy makers. The importance of innovation and innovative behavior of employees is imperative for the sustainability of organizations. The current study is conducted in an educational context where the faculty members are involved in teaching and research. First, the positive effect of PPAQ on employee innovative behavior directly and indirectly through psychological empowerment calls for special attention. Given that PPAQ promotes innovative behavior among faculty members, organizations should develop such appraisal practices that ensure quality. The supervisor/head of the department is responsible for improvements in teaching and research. It implies that an open communication policy must prevail and be ensured through regular feedback. The performance appraisal needs to be supportive for employees as it gives voice to the problems faced by the faculty members rather than practicing it as a monitoring mechanism. The discussion and feedback highlighting the improvement areas create perceptions of quality in performance appraisal among employees. This in turn implies that employee will respond positively by adapting the innovative behavior. 

Second, the organization needs to psychologically empower their faculty members. This implies that they are empowered to provide innovation in teaching and practice by adopting the new methods, techniques, and technology. It will ultimately lead to better performance of faculty members in terms of teaching and research. Innovation in teaching generates new ideas and insights which improves the performance of students and leads to improved organizational performance as well. Third, the higher authorities or policy makers should device policies to promote innovation and innovative behavior. The organization should provide assistance to employees with respect to technological change. Training and continuous learning must be the focus of policies in the field of education. The faculty members should be empowered to bring about positive change in their work, and organizations must recognize and reward them. This will affect the performance of colleagues, groups and units as well. Lastly, the policies should be same at all levels or organizational hierarchy. Especially with respect to HR policy, the HRM system should send distinct and consistent messages to employees. This will eradicate biasness and favoritism. The consensus regarding HR policies should be present at all levels of an organization. 

### 5.3. Limitations and Future Research

The current studies carried out possessed several limitations that offer opportunities for future research. First, we used a cross-sectional design. A longitudinal design is needed and could allow for data to be gathered and analyzed for the same or similar research to come closer to causality inference. Second, in the current study innovative behavior was studied and assessed as a unidimensional construct. The exploration of different dimensions of innovative behavior can be interesting for future research. It is suggested that separate assessments for creation, promotion, and implementation of new ideas should be made in future research. Similarly, the current study focused only on a single element of performance appraisal i.e. PPAQ. Future researchers should include other components of performance appraisal such as satisfaction with and effectiveness of performance appraisal in their studies. Third, future studies may explore the mediating role of other variables in the relationship between PPAQ and innovative behavior such as job satisfaction and affective commitment. In addition, the role of other moderating variables like organizational culture, perceived organizational support, and structural empowerment can also be explored in future studies. Lastly, the participants in this research were the faculty members working in public sector HEIs in Islamabad, Pakistan, which calls generalizability into question. Future studies may replicate the results of our study in other samples and contexts. 

### 5.4. Concluding Remarks

The current study empirically demonstrated that PPAQ has a positive impact on employee innovative behavior. The relationship between PPAQ and innovative behavior is mediated by psychological empowerment. Further we found that the effects of PPAQ on psychological empowerment and innovative behavior are moderated by the perceptions of HRM system strength. When employees perceive quality in performance appraisals, they tend to be more innovative at work by adopting new skills, techniques, and practices. The encouragement of, and provision for, faculty professional development and innovative behavior involves a re-thinking of the traditional approaches to performance appraisals. In order to stimulate innovative behavior from employees, managers should empower their employees and one way of doing so is to enhance their perceptions regarding the quality of performance appraisals. Employee innovative behavior is an important prerequisite for organizational growth, development, and sustainability and requires special attention from managers and policy makers, particularly in the context of higher education institutes. 

## Figures and Tables

**Table 1:** Results of the measurement model.

First-Order Constructs	Second-Order Constructs	Indicator’s	Factor Loading	CR	AVE	Convergent Validity
Perceptions of Performance Appraisal Quality		PQ1 PQ2 PQ3	0.883 0.869 0.891	0.912	0.776	Yes
Meaning		ME1 ME2 ME3	0.940 0.937 0.938	0.957	0.880	Yes
Competence		CO1 CO2 CO3	0.927 0.929 0.937	0.951	0.867	Yes
Self-Determination		SD1 SD2 SD3	0.936 0.938 0.934	0.955	0.876	Yes
Impact		IM1 IM2 IM3	0.950 0.956 0.948	0.855	0.664	Yes
	Psychological Empowerment	Meaning Competence Self-Determination Impact	0.894 0.898 0.881 0.877	0.937	0.788	Yes
Distinctiveness		DI1 DI2 DI3 DI4 DI5 DI6	0.853 0.845 0.841 0.880 0.823 0.852	0.939	0.721	Yes
Consistency		CT1 CT2 CT3 CT4 CT5 CT6	0.840 0.872 0.907 0.914 0.815 0.836	0.947	0.748	Yes
Consensus		CS1 CS2 CS3 CS4	0.922 0.921 0.922 0.920	0.957	0.848	Yes
	Perceptions of HRM system strength	Distinctiveness Consistency Consensus	0.915 0.916 0.908	0.938	0.834	Yes
Innovative Behavior		IB1 IB2 IB3 IB4 IB5	0.838 0.856 0.889 0.822 0.805	0.924	0.710	Yes

Note: CR = Composite Reliability; AVE = Average Variance Extracted.

**Table 2:** Means, Standard Deviations, and Correlations among constructs.

Constructs	Mean	SD	1	2	3	4	5	6	7	8	9
1. Perceptions of Performance Appraisal Quality	4.100	0.646	0.881								
2. Meaning ^a^	4.108	0.799	0.433	0.938							
3. Competence ^a^	4.092	0.780	0.424	0.788	0.931						
4. Self-Determination ^a^	4.025	0.821	0.376	0.695	0.717	0.936					
5. Impact ^a^	3.989	0.928	0.398	0.690	0.686	0.720	0.951				
6. Distinctiveness ^b^	4.373	0.596	0.065	0.047	0.010	0.036	−0.066	0.849			
7. Consistency ^b^	4.182	0.639	0.023	0.002	−0.032	0.009	−0.099	0.731	0.865		
8. Consensus ^b^	4.263	0.672	0.044	0.026	−0.027	0.001	−0.062	0.767	0.758	0.921	
9. Innovative Behavior	4.386	0.587	0.399	0.414	0.395	0.396	0.451	−0.083	−0.057	−0.051	0.843

*Note:*^a^ First-order constructs of the higher-order construct psychological empowerment. ^b^ First-order constructs of the higher-order construct perceptions of HRM system strength.
